# Seasonal Variations in 25-Hydroxyvitamin D Levels among Pediatric Patients Attending the Healthcare Centre

**DOI:** 10.3390/nu16030379

**Published:** 2024-01-27

**Authors:** Tarek Benameur

**Affiliations:** Department of Biomedical Sciences, College of Medicine, King Faisal University, Al Ahsa 31982, Saudi Arabia; tbenameur@kfu.edu.sa

**Keywords:** deficiency, insufficiency, health, healthcare, pediatric outpatients, prevalence, Saudi Arabia, seasonal impact, seasonality, vitamin D, 25-hydroxyvitamin D

## Abstract

Vitamin D plays an essential role in maintaining bone density, building the immune system, and regulating cell growth alongside other key biological functions. Limited data are available about the seasonal variation in vitamin D levels in the pediatric population in Saudi Arabia. This study aimed to investigate the seasonal influence on the pediatric circulating levels of 25(OH)D. A total of 1790 pediatric outpatients who visited the University healthcare centre were included in this study. Overall, there was a noticeably high prevalence (69%) of both combined 25(OH)D deficiency and insufficiency. The highest mean serum concentration of 25(OH)D was recorded in summer (29 ng/mL) and autumn (27 ng/mL). The deficient and insufficient categories were predominant, accounting for 33% and 36%, respectively. Comparable patterns were recorded during autumn, winter, and spring. Interestingly, the 25(OH)D level was significantly associated with the four seasons (*p* = 0.001), with females having a higher prevalence of 25(OH)D deficiency in the spring and summer than males. Furthermore, only in autumn and winter, we found a significant association between gender and 25(OH)D status (*p* < 0.001 for both). Another association between nationality and the circulating levels 25(OH)D was found during autumn and winter (*p* < 0.001 and *p* = 0.01), respectively. In all seasons, age had a negative impact on serum (OH)D levels. However, this relationship was statistically significant (*p* < 0.05) only in summer, autumn, and winter. Gender was a significant predictor, with 25(OH)D levels in autumn and winter and an odds ratio of 1.67 in autumn and 2 in winter, indicating that being men had a positive influence on circulating 25(OH)D levels. There were highly significant differences in 25(OH)D concentrations among different age categories. The Saudi population experiences low levels of vitamin D, particularly in autumn and winter periods. This study showed that seasonality, age category, nationality, and gender influence vitamin D status, suggesting the need for tailored intervention and monitoring of 25(OH)D status to reach adequate levels of vitamin D. Healthcare practitioners and policymakers may consider the interplay between age, nationality, gender, and seasonal variations when addressing vitamin D status and a targeted supplementation approach for high-risk groups that may develop health issues.

## 1. Introduction

Vitamin D (1,25 dihydroxyvitamin D3) is synthesized endogenously from 7-dehydrocholesterol within the epidermis in response to UVB irradiation and can be obtained from dietary sources and supplements [1,2].

Various international societies have proposed different cut-off sets for identifying vitamin D deficiency in the pediatric population. At present, the European Society of Pediatric Gastroenterology, Hepatology and Nutrition (ESPGHAN) [3], the Endocrine Society [4], and the Society for Adolescent Health and Medicine [5] have proposed that serum 25(OH)D levels falling within the range of 0–20 ng/mL should be classified as deficient, from 21 to 29 ng/mL, as insufficient, and a level of ≥30 ng/mL as sufficient.

Vitamin D is known for its multifaceted role in maintaining overall health and well-being [6]. In addition to its various physiological functions, vitamin D is crucial for the regulation of calcium and phosphorus homeostasis, which has a direct impact on bone growth and mineralization [7]. Beyond its role in stabilizing endothelial cell membranes, vitamin D exerts immunological effects on multiple immune system functions.

Based on growing evidence, an association between vitamin D deficiency and increased risk of developing several immune-related disorders, including psoriasis, type 1 diabetes, multiple sclerosis, rheumatoid arthritis, tuberculosis, sepsis, cancer, cardiovascular diseases, respiratory tract infection and COVID-19, has been observed [4,8,9,10,11,12,13,14,15,16,17].

As was previously reported, vitamin D has significant implications for the pediatric population’s health and development [18]. This highlights how its effects extend beyond the maintenance of skeletal homeostasis. Despite being a global health concern affecting individuals of all ages, vitamin D deficiency exerts a detrimental effect on the pediatric population, primarily due to the accelerated growth and critical developmental milestones during childhood and adolescence [19]. Recent studies have focused on the association between insufficient levels of vitamin D and a wide range of adverse health outcomes in children, including low bone mineral density, rickets, osteomalacia, delayed growth, a short stature, compromised immune function, and an increased risk of various chronic diseases later in life, such as autoimmune disorders, cardiovascular diseases, and other diseases [20,21,22,23,24]. Severe vitamin D insufficiency may also be associated with hypocalcemia, which can result in tetany, seizures, and infant heart failure [25].

Recent investigations have revealed that the prevalence of vitamin D deficiency remains alarmingly high among pediatric populations worldwide. Notably, in countries with limited sun exposure, like Finland and Norway, during winter, it was reported that around 70% of children were found to have insufficient vitamin D levels [26]. Similarly, [27] found that 45% of adolescents had alarmingly high rates of low vitamin D levels. The prevalence of vitamin D deficiency has also been reported to reach 70% in studies carried out in highly polluted areas, such as urban areas in China and India [28,29].

The incidence and prevalence of this condition among the pediatric population remain alarmingly high, with several contributing factors, such as inadequate sun exposure, dietary habits, air pollution, latitude, geographical location, the use of sunscreen, and a sedentary attitude influencing its prevalence in different regions [30].

Several factors have been identified as risk factors contributing to lower vitamin D levels, including age, gender, traditional attire, deliberate sun avoidance, insufficient dietary intake, a hot climate, and reduced outdoor activities, as reported in reference [31].

These findings emphasize the critical importance of understanding the factors that contribute to vitamin D deficiency in the pediatric population, with seasonal variations emerging as a salient aspect of this complex issue. In recent years, an increasing body of scientific research has shed light on the profound effects of seasonal variations on pediatric vitamin D status, presenting both challenges and opportunities in the quest to address this nutritional deficiency [32].

Seasonal variations in vitamin D status are particularly pronounced in regions with limited sunlight exposure during certain periods of the year. The eastern region of Saudi Arabia experiences extreme hot climate conditions with scorching summers and mild winters, which can impact the pediatric population’s exposure to sunlight for vitamin D synthesis. Recent research has revealed significant seasonal fluctuations in vitamin D levels among the young population, with lower levels observed during summer due to avoidance of sun exposure and increased indoor activities to escape the heat and higher levels during milder winter conditions when outdoor activities are more common [33]. These findings underscore the importance of considering regional variations and climate conditions when addressing pediatric vitamin D deficiency.

Taken together, we postulate that seasonal effects could lead to inadequate serum levels of vitamin D in the study population and might indirectly lead to an increased incidence of medical problems associated directly or indirectly with vitamin D status. However, data on the association between seasonal effects and vitamin D status in pediatric patients in Saudi Arabia are lacking. Hence, the aims of this study were as follows:

- Investigate whether seasons have an impact on vitamin D concentrations and determine the 25(OH)D trend and seasonal variations in serum 25(OH)D levels.
- Identify the predictors of this change in pediatric patients visiting polyclinics.

This research can help validate this hypothesis and encourage the implementation of necessary public health measures to address the health concerns among the developing pediatric population.

## 2. Materials and Methods

### 2.1. Study Design and Settings

This retrospective chart review aimed to explore pediatric patients’ medical records attending the outpatient department of the healthcare centre at King Faisal University in Al-Ahsa, Saudi Arabia. This study focused on the period between September 2019 and February 2021.

### 2.2. Participants

Data from 1790 individuals were extracted from the electronic health records system of the University healthcare centre. The study included a paediatric population aged between 0 and 21 years of either gender visiting the health care centre for a health examination and who had their serum vitamin D concentrations measured at least once within the study period.

### 2.3. Biochemical Analysis

Serum 25(OH)D levels were determined using a radioimmunoassay technique (Roche Diagnostics, Mannheim, Germany), and blood samples were obtained from outpatients who visited the healthcare centre. Due to its relatively long half-life, serum 25(OH)D concentrations, rather than 1, 25(OH)D concentrations, were the primary recognized indicator of vitamin D status, representing endogenously produced vitamin D and vitamin D obtained through diet and supplementation [34]. The assay’s precision, accuracy, calibration, and comparison with the standardized measurement were established according to the manufacturer’s protocol, as previously described [35,36].

### 2.4. Outcome Measures

The primary outcome of this study was centered on assessing serum 25(OH)D levels and determining the percentage of pediatric outpatients with vitamin D insufficiency, deficiency, and sufficiency as per the Endocrine Society recommendations described above. In addition to this primary outcome, we also examined several secondary outcome measures, including the relationship between serum 25(OH)D levels and a child’s age, the prevalence of vitamin D deficiency categorized by gender and nationality, the seasonal variations in 25(OH)D levels, and the cumulative frequency distribution of serum 25(OH)D among the pediatric population visiting the healthcare centre.

### 2.5. Statistical Analysis

The extracted data were analyzed using an SPSS 26.0 software package, IBM SPSS Statistics for Windows (IBM Corp., Armonk, NY, USA). The serum 25(OH)D levels are shown as the means and standard deviations (±SD), medians, and interquartile ranges. Absolute values and relative frequencies (percentages) are used to represent categorical variables.

The seasons for blood sample collection periods were categorized as follows: spring (21 March to 20 June), summer (21 June to 22 September), autumn (23 September to 20 December), and winter (21 December to 20 March). The mean serum 25(OH)D levels were compared between different seasons (Autumn, winter, spring, and summer), using Student’s *t*-test (for normally distributed data), the Mann–Whitney U-test (for non-normally distributed data) or one-way analysis of variance (ANOVA).

The differences in frequencies and association between variables were tested with Pearson’s Chi-square test. Factors affecting vitamin D were determined using a linear regression analysis. Gender, age, nationality, and the date of blood sample collection were among the variables for which a correspondence analysis was conducted. Additionally, within each season, the association between vitamin D levels and sociodemographic characteristics, such as age, gender, and nationality, was examined using a linear regression analysis.

Frequencies and percentages (%) were reported for categorical variables. Tests to determine the differences in mean serum 25(OH)D levels via sex, age, and season were performed using analysis of variance. We used logistic regression to examine whether the prevalence of vitamin D insufficiency or deficiency differed by sex, age, or season as a predictor. Statistical significance was set at *p* < 0.05.

## 3. Results

### 3.1. The Overall 25(OH) Vitamin D Status and Pediatric Patients’ Characteristics

Table 1 provides a comprehensive analysis of the population characteristics and the distribution of the serum concentration of 25(OH)D status among individuals. The study population comprises 1790 individuals with an average age of 16.23 ± 5.44 (SD) (median age of 19). The segmentation across age categories shows intriguing trends as follows: infancy (0–1 year) is characterized by a marginal presence of 0.3%, followed by early childhood (2–5 years) at 7% and 6–11 for middle childhood at 14% with a substantial majority during late adolescence (19–21 years), constituting a proportion of 987 (55%).

The gender distribution shows that 75% of the participants were female, while 25% were male. In terms of nationality, the majority (79%) were Saudi nationals, with 21% being non-Saudi. In terms of the serum 25(OH)D status, most of the population fell into the ‘deficient’ and ‘insufficient’ categories, irrespective of age, gender, or nationality.

### 3.2. Serum 25(OH) Levels in Pediatric Patients Aged 0–21 Years, Stratified by Outpatient Characteristics

Table 2 presents findings related to the 25(OH)D serum concentration stratified by various outpatient characteristics and seasons of blood sample collection. The comparison of the means of serum 25(OH)D showed that male outpatients had a significantly higher mean 25(OH)D level (29 ng/mL) when compared to female outpatients (26 ng/mL) (*p* < 0.001). Despite the recorded significant difference in the average serum concentrations of 25(OH)D between groups, this confirmed an insufficient status among the individuals.

Age groups also showed significant variations (*p* < 0.001), with infants (0–1) and early childhood (2–5) displaying higher mean concentrations of serum 25(OH)D (66 ng/mL and 40 ng/mL) than other age categories. Overall, there was a statistically significant difference in mean 25(OH)D levels among age groups (*p* < 0.001).

In this cohort, Saudi patients had a statistically significant lower mean of 25(OH)D level (26 ng/mL) compared to non-Saudi patients (29 ng/mL) (*p* < 0.001).

The seasonal variations in serum 25(OH)D levels show that the highest mean serum concentration of 25(OH)Dwas recorded in summer (29 ng/mL), followed by autumn (27 ng/mL), spring (25 ng/mL), and winter (25 ng/mL). It is crucial to emphasize that despite seasonal variations in mean concentrations, all recorded mean serum concentrations of 25(OH)D for each season fall within the ‘insufficient’ level.

This demonstrates the influence of seasonal variation on serum 25(OH)D levels, highlighting the importance of sunlight exposure as a determinant of vitamin D status. Furthermore, statistically significant differences in serum 25(OH)D levels based on gender, age groups, nationality, and the season of blood samples were observed.

**Figure 1:**
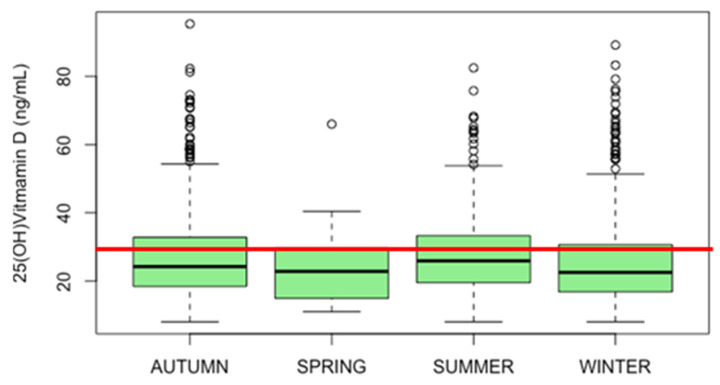
Seasonal fluctuation in the median serum 25(OH)D distribution of the pediatric population. The red line indicates a sufficient level of 30 ng/mL.

Figure 1 illustrates the seasonal fluctuation in the median serum 25(OH)D distribution. Median values are 24 ng/mL in autumn, 23 ng/mL in spring, 26 ng/mL in summer, and 23 ng/mL in winter. The lowest levels of 25(OH)D were registered in autumn, summer, and winter with 8 ng/mL each. The summer period shows the highest median serum 25(OH)D level of 26 ng/mL with an IQR of 14 for all seasons except spring, which shows the highest IQR of 15.

The data were expressed as ng/mL. The median values were 24 ng/mL in autumn, 23 ng/mL in spring, 26 ng/mL, and 23 ng/mL in winter. The lowest levels of 25(OH)D were registered in autumn, summer, and winter with 8 ng/mL each. The summer period showed the highest median serum 25(OH)D level of 26 ng/mL with an IQR of 14; the highest IQR was recorded in the spring (15). In all seasons, the participants presented a state of 25(OH)D deficiency (0–20 ng/mL). A comparison of means using the ANOVA test shows a statistically significant difference between groups (*p* = 0.007).

Table 3 demonstrates the seasonal variation in the 25-hydroxyvitamin D status of the study population. Individuals were categorized into three distinct groups based on their 25(OH)D levels as follows: sufficient (≥30 ng/mL), insufficient (21–29 ng/mL), and deficient (0–20 ng/mL).

A notable seasonal variation in serum 25(OH)D was recorded. During spring, a higher proportion of individuals fell into the deficient category (45%), whereas only 25% were categorized as “sufficient”. A comparable pattern was recorded during winter, with the deficient category being the most prevalent (42%), followed by the insufficient category (31%).

However, during summer, the sufficient category dominated, representing (38%) of the population, followed by “insufficient” (35%), indicating a seasonal improvement in vitamin D status. Autumn exhibited a more balanced distribution among the three categories, with 35% categorized as “insufficient”, and 33% as “deficient”.

Remarkably, we found that there is an association between the outpatients’ circulating levels of 25(OH)D and the four seasons (*p* = 0.001). These findings underscore the presence of seasonal variation in 25(OH) vitamin D status within the study population.

Taken together, vitamin D insufficiency and deficiency prevailed across all seasons in the studied population, emphasizing the need for consistent strategies to address this issue.

Table 4 provides a comprehensive overview of the distribution of 25(OH) vitamin D levels within different seasons stratified by gender. During spring, the prevalence of 25(OH)D deficiency was higher in females (35%) when compared with male individuals (10%). Similarly, during summer, a higher prevalence of 25(OH)D deficiency was also observed in female outpatients (22%). Similar patterns were found in autumn and winter, where a higher prevalence of 25(OH)D deficiency was observed in female outpatients (29%), (36%), respectively.

This statistical analysis indicates an association between gender and the status of 25(OH)D in autumn and winter (*p* < 0.001, *p* < 0.001), respectively. However, gender is less likely to affect 25(OH)D status during spring and summer (*p* = 0.966, *p* = 0.139), respectively.

### 3.3. 25-Hydroxyvitamin D Status Stratified by Nationality

As illustrated in Table 5, the comparison of a mean serum concentration of 25(OH)D between Saudi and non-Saudi outpatients showed significant differences in spring, autumn, and winter (*p* = 0.04, *p* = 0.04, *p* = 0.006), respectively. However, in summer, there was no significant difference in the mean serum concentration of 25(OH)D between Saudi and non-Saudi outpatients (*p* = 0.170).

It is noteworthy to emphasize that Saudi outpatients consistently exhibited insufficient 25(OH)D levels across all seasons, whereas non-Saudi patients displayed sufficient levels only during the spring and summer periods.

As depicted in Table 6, the prevalence of 25(OH)D deficiency was significantly higher in Saudi outpatients when compared to non-Saudi outpatients for all seasons, including spring (35% vs. 10%), summer 22% vs. 6%), autumn (30% vs. 4%) and winter (36 vs. 5).

These results show a significant association between nationality and the status of circulating 25(OH)D, particularly during the autumn and winter seasons when blood samples were collected (*p* < 0.001 and *p* = 0.01), respectively.

In Table 7, the linear regression analysis estimates the impact of age and nationality on the circulating levels of 25(OH)D in outpatients visiting the healthcare centre for the four seasons. Linear regression analysis found that age affected the serum 25(OH)D levels negatively in all seasons. However, this relationship is statistically significant (*p* < 0.05) only in summer, autumn, and winter.

### 3.4. 25(OH) Vitamin D Status Stratified by Gender

In line with the findings described in (Table 4), as shown in Table 8, the binary logistic regression analysis confirmed that there is a statistically significant association between gender and 25(OH)D levels in autumn and winter (*p* = 0.001, *p* < 0.001), respectively. Being male had a significant positive impact on the circulating level of 25(OH) vitamin D in autumn and winter, whereas this impact disappeared during spring and summer.

As illustrated in Table 8, the odds ratio is 1.67, indicating that male outpatients are 1.67 times more likely to have sufficient 25(OH)D levels compared to females during autumn. In winter, this chance increased to 2. However, in the spring and summer seasons, this chance decreased to 1 and 1.09 (respectively).

In conclusion, gender is a significant predictor during autumn and winter, with male outpatients more likely to have sufficient levels of 25(OH)D levels compared to female outpatients.

As shown in Table 9, there was a significant seasonal variation in the 25(OH)D levels between all age groups. The highest serum concentrations were recorded during winter, with the highest mean value of 71 ng/mL among infants aged between 0 and 1. Conversely, the lowest means were observed in spring, with the lowest mean concentration of 20 ng/mL in the 12–18 age group. There were highly significant differences in 25(OH)D concentrations between different age categories.

Additionally, we found a statistically significant association between age categories and the 25(OH) vitamin D status in autumn, winter, and summer (*p* < 0.001, 0.001 and *p* < 0.001), respectively. Surprisingly, no association was found in spring.

## 4. Discussion

To the best of our knowledge, this is the first study in Saudi Arabia exploring the seasonal changes in 25(OH)D serum levels in the pediatric population in the eastern region of Saudi Arabia. Herein, we provided novel insight into the impact of seasons on the 25(OH)D serum levels of the pediatric population visiting the healthcare centre, which was measured in the healthcare centre laboratory. We compared the concentration of the circulating 25(OH)D in pediatric sera for spring, summer, autumn, and winter based on the conservative cutoff point suggested by the Endocrine Society. Gender, nationality, and age were also investigated as predictors of change in 25(OH)D levels.

Low levels of vitamin D are emerging as a major global public health problem occurring worldwide and mainly in Asia, including the Middle East and North African countries [37,38]. Indeed, vitamin D deficiency and its associated risk factors in the Middle East and Saudi Arabia are common in both adults and children [33].

However, the prevalence varies significantly across countries and subpopulations due to changes in risk factors, including skin pigmentation, sun exposure, lifestyle, and environmental factors; prenatal risk factors, such as maternal VDD and premature birth; postnatal risk factors, such as prolonged breastfeeding, low dietary intake, dark skin pigmentation or low sun exposure; and obesity. Other risk factors include malabsorptive conditions, genetic disorders, medications like certain anticonvulsants, anti-fungal agents, and glucocorticoids, in addition to some chronic diseases, such as kidney disease, liver disease, etc. [39,40,41].

Furthermore, in adults, low levels of vitamin D were predicted for advanced age, the female gender, multiparity, clothing style, season, socioeconomic position, low calcium intake or vitamin D supplement, low physical activity, and urban residence [42,43].

Recent evidence from the literature has reported high variations in vitamin D deficiency within the different WHO regions, with the highest prevalence recorded in the eastern Mediterranean region at a latitude between 20 and 50° north for countries like Bahrain (49.4%), Iraq (31.1%) and Saudi Arabia (37.4%) which record a high prevalence of VDD. Alarmingly, VDD was observed in 58.9% of the Kuwait population who were aged 10 [44,45]. The present study’s findings are consistent with the recent literature confirming a high prevalence of vitamin D deficiency or insufficiency in the pediatric population.

The major findings of this study are as follows: (i) Despite the seasonal variations, the mean and the median of the serum concentrations of 25-(OH) D within the four seasons fall within the insufficient group, with men showing a significantly higher median than female outpatients. (ii) Significant differences between age groups were found, with only infants (0–1) and early childhood (2–5) displaying sufficient levels when compared to other age groups. (iii) The highest mean serum concentration of 25(OH)D was recorded in the summer, followed by autumn (iv) There is an association between the circulating levels of 25(OH)D and the four seasons, where almost half of the outpatients fall within the deficient category in spring, with a comparable proportion in winter (v). Another association between gender and the 25(OH)D status in autumn and winter was found. (vi) Gender affected the 25(OH) concentrations both positively and significantly. In comparison to females, male pediatric patients have a 1.67 chance of sufficient levels of 25(OH)D during autumn. In winter, this likelihood increases to two.

However, this chance drops to 1 in summer and spring. Only during autumn and winter does being a male have a significant positive impact on vitamin D levels.

(vii) A significantly higher prevalence of vitamin D deficiency among Saudis when compared to non-Saudi outpatients in all seasons was found. (viii) Nationality was shown to be associated with the status of circulating 25(OH)D only during autumn and winter. (ix) Age negatively and significantly affected the circulating 25(OH)D levels during summer, autumn, and winter.

Despite the sunny weather in the eastern region throughout the four seasons in Saudi Arabia, our data demonstrate a clear seasonal trend, with higher means of serum levels of 25(OH)D in summer and autumn compared to winter and spring. This retrospective chart review, encompassing a representative sample of the pediatric population, clearly demonstrates a high prevalence of combined 25(OH)D insufficiency and deficiency. In fact, 69% of the study cohort exhibited levels below the recommended cutoff of 30 ng/mL. This is comparable to the regional and global-recorded prevalence of vitamin D deficiency in Saudi Arabia [46]. However, the proportion of vitamin D deficiency among children and adolescents remains lower when compared to previous studies [42].

In this study, the proportion of the pediatric population with 25(OH)D deficiency was higher in spring and winter when compared to summer and autumn, suggesting a seasonal trend of 25(OH)D change. This could be attributed to a combination of environmental, dietary, and lifestyle factors. The key possible reasons are as follows: reduced sun exposure, the zenith angle of the sun leading to fewer UVB photons reaching the earth’s surface during winter and spring, and geographical location and latitude [47]. Indeed, the effective absorption of ultraviolet B (UVB) radiation for the synthesis of vitamin D is facilitated by the lower solar zenith angle [48,49,50]. Additionally, a sedentary lifestyle and poor dietary intake of vitamin D can be considered as well [51].

Additionally, at this age period, this population has a tendency to use electronic devices indoors rather than engaging in outdoor physical activities and playing; parents’ have lower awareness about sedentary behavior and its impact not only on the incidence of obesity and its associated health outcomes but also on the levels of vitamin D. Thus, sun exposure is limited [52].

The highest prevalence of 25(OH)D deficiency and insufficiency recorded in winter and autumn among the population aged between 19 and 21 when compared to the younger population is corroborated by previous studies [53]. As previously described, maternal 25(OH) levels can be also considered a risk factor for neonates or infants [41]. This requires further investigation to confirm this hypothesis. In addition to the above-mentioned risk factors for low levels of 25(OH)D among the young population, other factors may interfere as well, such as a lack of vitamin D supplementation, dietary habits and quality that reflect individual, cultural preferences, education, socioeconomic background, and health status. According to the recent literature, adolescents have a tendency to follow an unhealthy diet [54], with a lower intake of vitamin D-fortified dietary products. A poor fish-enriched diet (salmon), as well as social habit changes, such as a preference for fast food, and over-cooked vitamin D-enriched food also can be considered. Aside from their addiction to the digital world, which has a direct impact on their time spent outdoors for physical activities [34,55,56], we also suggest that an unhealthy diet can have a negative impact on 25(OH)D levels in the younger population, though to a lesser extent. When considering the findings of our study, along with previous research, we recommend the consumption of vitamin D-fortified food to address vitamin D deficiency, particularly in the pediatric population, given its critical role in bone growth, development, and the prevention of certain diseases.

The current study found that the highest serum 25(OH)D levels were recorded in summer and autumn. This association between seasonal changes and vitamin D status has also been addressed in several previous studies, some of which are from mid-latitude areas. These findings support the research findings [57,58,59,60,61]. We suggest that this difference between seasons can be corrected to some extent by increasing the time of sun exposure and vitamin D supplementation to maintain sufficient levels of vitamin D [62].

Among the reasons for the increasing prevalence of vitamin D insufficiency or deficiency in the pediatric population reported in the literature, a lack of knowledge and awareness about vitamin D deficiency and preventive measures such as vitamin D supplementation is highlighted [42,46,63]. During autumn and winter, the percentage of female pediatric patients with deficiency is higher than men in winter and autumn. This may be explained by insufficient knowledge about vitamin D, the frequent use of sunscreen and full clothing style, and the limited sun exposure among female patients consistent with the reported findings by [64].

A recent study in Saudi Arabia revealed a high prevalence of Vitamin D deficiency among infants aged up to two years in the southwestern region of Saudi Arabia. The major independent risk factors of VDD identified in this study were the place of residence, the duration of sun exposure not exceeding 3 days per week and being breastfed only [65]. Although our results are slightly different from these results, the risk factors described above would be similar.

These differences could be due to the population size being smaller in our study than in other age groups, in addition to the unavailability of vitamin D supplementation status in infancy. Thus, drawing a conclusion for this category requires additional research with a larger number of participants in this age range and investigating the aforementioned risk factors.

Our findings indicate that gender has a significant impact on the prevalence of decreased levels of 25(OH)D during autumn and winter, which is in accordance with earlier research associating the female gender with vitamin D deficiency or insufficiency [66].

When investigating nationality as a predictor of 25(OH)D seasonal changes, we found that Saudi patients have a significantly higher prevalence of 25(OH)D deficiency than non-Saudi pediatric patients. This appears to be consistent with other previous research data, regardless of the place of residency or the diet [66,67]. Indeed, the investigation of factors influencing serum levels of 25(OH)D in various studies suggests a strong genetic influence on serum concentrations [68,69].

This may explain the differences between Saudi and non-Saudi patients. Factors other than diet and UV exposure may also affect the 25(OH)D concentrations, such as genetic factors as previously described [70]. Additionally, a recent study found that the GG allele of the three SNPs, GC rs4588, CYP2R1 rs10741657, and VDR rs2228570, is present in patients with significantly lower levels of 25(OH)D compared to those with normal levels [71]. Other factors were not investigated in this research, including vitamin D hydroxylase, the vitamin D-binding protein, and the inactivation of 25(OH)D-24-hydroxylase by cytochrome P450, CYP24, and CYP3A4. This could be one of the elements causing differences in vitamin D levels between various populations [72,73]. It is worth noting that non-Saudi patients originated from at least 24 different countries with different ethnic backgrounds. However, the impact of non-Saudi ethnicity/race on the serum levels of vitamin D was not investigated in this study.

Previous research has shown an association between plasma 25(OH)D concentrations and vitamin D consumption, underscoring the importance of dietary vitamin D as a factor influencing plasma 25(OH)D concentrations [74]. Despite the lack of data on vitamin D intake and dietary habits among the study population, this factor may also explain the observed differences.

When considered collectively, these findings shed light on the multifactorial prevalence of low levels of vitamin D in the Saudi pediatric population during the various seasons. Age, gender, dress codes, cultural customs, high skin melanin content, vitamin D supplementation, exposure to sunlight, dietary habits, and vitamin polymorphism were all included [75,76,77]. To demonstrate to what extent genetic variations contribute to low levels of 25(OH)Din Saudi populations, further investigations are needed. Other factors were not available in this study and may be considered in discussing our results, such as self-supplementation with vitamin D, recent sun-rich periods, night shift workers, regular sunscreen use (that blocks the UVB), and religious and cultural dressing practices [78]. Furthermore, as previously described, BMI is often associated with lower levels of vitamin D [79]. Additionally, recent studies have reported a high prevalence of overweight and obesity among the Saudi pediatric population [80,81,82,83,84]. In a large cohort study, the prevalence of vitamin D deficiency and insufficiency was remarkably high in school-aged children with a high BMI, not only in Saudi Arabia [85]. This underscores the negative effects of obesity on overall children’s health, including inflammation, insulin resistance, impaired bone mineralization, osteoporosis, cardiovascular disease, and an increased risk of developing type 2 diabetes [86]. This provides further support for our findings related to this population.

Even though no data were obtained regarding clothing practices in this study, we believe that Saudi male and female dressing practices covering most of the skin may have led to altered vitamin D levels, particularly in the adolescent population. These factors are likely to influence the quantity of solar exposure regardless of the length of time spent outside. Female patients are more likely to cover their whole body and heads in public; hence, sun avoidance is more widespread in women.

It is worth noting that the observed impact of gender on vitamin D levels could potentially be attributed to the difference in skin pigmentation between male and female patients. Previous research has demonstrated a strong correlation between skin reflectance and both absolute latitude and UV radiation intensity [87]. This suggests that the primary function of melanin pigmentation in humans is to control how UV radiation affects the contents of cutaneous blood vessels found in the dermis. Although skin reflectance for this study is not available, we hypothesize that female patients may have lighter skin than male patients.

VDD and vitamin D insufficiency were shown to be associated with a variety of childhood disorders, including precocious puberty and dental caries. Furthermore, compared to normal subjects, recent studies have shown that precocious puberty subjects had lower vitamin D levels. This suggests that VDD increases the risk of precocious puberty [88,89].

Vitamin D was shown to prevent oral health disorders among the young population, particularly dental caries, which are a major public health problem among the pediatric population and remain alarmingly high in Saudi children compared to the global average. Several factors influencing the prevalence of dental caries in primary and permanent dentition in children have been identified. This includes socioeconomic status, their place of living, oral hygiene practice, diet, and vitamin D intake [90,91,92]. In children, VDD is considered a risk factor for primary and/or permanent dental caries. This results in defective teeth mineralization as well as defects in enamel and dentin, resulting in increased dental caries. Taken together, maintaining optimal levels of vitamin D during pregnancy and childhood can be considered a preventive measure for dental caries and promoting good oral health and development [93,94].

In line with the European guidelines and according to the existing literature demonstrating effective prevention of VDD in children aged 3 years and below [95,96], we suggest vitamin D supplementation at this age or when required. We warn against promoting the improper use of over-the-counter forms of native vitamin D, given the increased risk of intoxication. Nevertheless, it is important to provide children, if required, with native vitamin D and ensure they have sufficient dietary calcium to prevent rickets and maintain optimal bone health and bone density, particularly among the growing pediatric population [97].

Promoting physical activity should also be part of a shared clinical decision-making process involving children and their parents. Taken together, this emphasizes the importance of addressing and remedying this insufficiency throughout the year to achieve an optimal level.

## 5. Conclusions

This study’s findings emphasize the significantly high prevalence of vitamin D insufficiency and deficiency within the investigated population, further accentuated by notable seasonal fluctuations. This highlights the critical importance of tailored intervention and vigilant monitoring, particularly during seasons when serum 25(OH) vitamin D levels tend to be lower, to address the potential health impact associated with vitamin D insufficiency, especially among a growing population. In addition, gender differences were not significant during spring and summer but became highly significant during autumn and winter. This suggests that healthcare professionals and policymakers should consider the interplay between not only gender but also age and nationality, along with seasonal variations, when assessing and addressing vitamin D status in clinical practice and public health interventions.

## Figures and Tables

**Table 1:** Basic demographic characteristics of the study population.

		Serum 25(OH)D Status (ng/mL)			
		Total	Sufficient	Insufficient	Deficient
		n (%)	≥30 ng/mL	21–29	0–20
**Age**	0–21	1790 (100)	554 (31%)	591 (33%)	645 (36%)
**Age categories**	0–1 (Infancy)	5 (0.3)			
	2–5 (Early childhood)	117 (7)			
	6–11 (Middle childhood)	247 (14)			
	12–18 (Early adolescence)	434 (24)			
	19–21 (Late adolescence)	987 (55)			
**Mean (age) ± SD**		16.23 ± 5.44			
**Gender**					
	Male	441 (25)			
	Female	1349 (75)			
**Nationality**					
	Saudi	1412 (79)			
	Non-Saudi	378 (21)			

Age classification of the pediatric population according to the NICHD.

**Table 2:** Serum 25(OH) levels in the pediatric patients aged 0–21 years, stratified by outpatient characteristics.

Variables			25(OH)D Serum Concentration (ng/mL)		*p*-Value
		N (%)	Mean ± SD	Median (IQR)	
**Gender**	**Male**	441 (25)	29 ± 13	28 (12)	a: *p* < 0.001
	**Female**	1349 (75)	26 ± 12	23 (14)	
**Age classes**	**0–1**	5 (0.3)	66 ± 7	65 (11)	b: *p* < 0.001
	**2–5**	117 (7)	40 ± 12	39 (13)	
	**6–11**	247 (14)	28 ± 8	28 (11)	
	**12–18**	434 (24)	24 ± 11	22 (13)	
	**19–21**	987 (55)	25 ± 13	22 (13)	
**Nationality**	**Saudi**	1412 (79)	26 ± 13	23 (14)	a: *p* < 0.001
	**Non-Saudi**	378 (21)	29 ± 10	27 (13)	
**Season of** **blood sample** **collection**	**Spring**	20 (1)	25 ± 13	29 (15)	b: 0.007
	**Summer**	228 (13)	29 ± 13	26 (14)	
	**Autumn**	814 (45)	27 ± 12	24.2 (14)	
	**Winter**	728 (41)	26 ± 13	23 (14)	

Notes: The comparison of groups means 25(OH)D were tested using the following: a: independent *t*-test, b: one-way ANOVA. Abbreviations: 25(OH)D: serum 25-hydroxyvitamin D. Differences were tested using analysis of variance. IQR = interquartile ranges, SD = standard deviation.

**Table 3:** The 25-hydroxyvitamin D status of the study population during different seasons.

	25(OH)D Status Frequency n (%)	Sufficient (≥30 ng/mL) n = 554 (31)	Insufficient (21–29 ng/mL) n = 591 (33)	Deficient (0–20 ng/mL) n = 645 (36)
**Season**	**Spring**	5 (25)	6 (30)	9 (45)
	**Summer**	86 (38)	80 (35)	62 (27)
	**Autumn**	262 (32)	281 (35)	271 (33)
	**Winter**	201 (28)	224 (31)	303 (42)
	***p*** **-value**	0.001	0.001	0.001

Notes: Differences in frequencies were tested using the Chi-square test.

**Table 4:** Seasonal variations in 25(OH) vitamin D status stratified by gender.

			25(OH) Vitamin D Serum Concentrations (ng/mL)				
Seasons			Sufficient	Insufficient	Deficient	Total	*p*-Value
**Spring**	**Female**	% within gender	4 (25%)	5 (31%)	7 (44%)	16 (100%)	0.966
		% of Total	20%	25%	35%	80%	
	**Male**	% within gender	1 (25%)	1 (25%)	2(50%)	4(100%)	
		% of Total	5%	5%	10%	20%	
	Total	% within gender	5 (25%)	6 (30%)	9 (45%)	20 (100%)	
**Summer**	**Female**	% within gender	59 (37%)	51 (32%)	49 (31%)	159(100%)	0.139
		% of Total	26%	22%	22%	70%	
	**Male**	% within gender	27 (39%)	29 (42%)	13 (19%)	69 (100%)	
		% of Total	12%	13%	6%	30%	
	Total	% within gender	86 (38%)	80 (35%)	62(27%)	228 (100%)	
**Autumn**	**Female**	% within gender	182(29%)	204 (33%)	233 (38%)	619 (100%)	*p* < 0.001
		% of Total	22%	25%	29%	76%	
	**Male**	% within gender	80 (41%)	77 (39%)	38 (20%)	195 (100%)	
		% of Total	10%	9%	5%	24%	
	Total	% within gender	262 (32%)	281 (35%)	271 (33%)	814 (100%)	
**Winter**	**Female**	% within gender	134 (24%)	159 (29%)	262 (47%)	555 (100%)	*p* < 0.001
		% of Total	18%	22%	36%	76%	
	**Male**	% within gender	67 (39%)	65 (38%)	41 (24%)	173 (100%)	
		% of Total	9%	9%	6%	24%	
	Total	% within gender	201 (28%)	224 (31%)	303 (41%)	728 (100%)	

Differences between genders were tested using the Chi-square test.

**Table 5:** The comparison of 25(OH) vitamin D means between Saudi and non-Saudi outpatients for the four seasons.

		Nationality	N (Frequency)	Mean of 25(OH)D Concentrations (ng/mL)	Std. Deviation	*p*-Value
**Spring**	25(OH)D	Non-Saudi	8	31	16	0.047
		Saudi	12	20	7	
**Summer**	25(OH)D	Non-Saudi	62	30	12	0.170
		Saudi	166	28	14	
**Autumn**	25(OH)D	Non-Saudi	169	28	9	0.048
		Saudi	645	27	13	
**Winter**	25(OH)D	Non-Saudi	139	28	11	0.006
		Saudi	589	25	13	

The comparison of mean 25(OH) vitamin D between Saudi and non-Saudi patients were tested using the independent *t*-test. Abbreviations: 25(OH)D: serum 25-hydroxyvitamin D, SD: standard deviation.

**Table 6:** Seasonal status of 25(OH) vitamin D stratified by nationality.

Seasons				25(OH) Vitamin D Serum Concentrations (ng/mL)				
				Sufficient	Sufficient	Deficient	Total	*p*-Value
**Spring**	**Nationality**	**Non-Saudi**	% within Nationality	4 (50%)	2 (25%)	2(25%)	8 (100%)	0.09
			% of Total	20%	10%	10%	40%	
		**Saudi**	% within Nationality	1 (8.3%)	4 (33.3%)	7 (58.3%)	12 (100%)	
			% of Total	5%	20%	35%	60%	
	Total		% within Nationality	5 (25%)	6 (30%)	9 (45%)	20 (100%)	
			% of Total	25%	30%	45%	100%	
**Summer**	**Nationality**	**Non-Saudi**	% within Nationality	31 (50%)	18 (29%)	13 (21%)	62 (100%)	0.06
			% of Total	14%	8%	6%	27%	
		**Saudi**	% within Nationality	55 (33%)	62 (37%)	49 (30%)	166 (100%)	
			% of Total	24%	27%	22%	73%	
	Total		% within Nationality	86 (38%)	80 (35%)	62 (27%)	228 (100%)	
			% of Total	38%	35%	27%	100%	
**Autumn**	**Nationality**	**Non-Saudi**	% within Nationality	66 (39%)	74 (44%)	29 (17%)	169 (100%)	*p* < 0.001
			% of Total	8%	9%	4%	21%	
		**Saudi**	% within Nationality	196(30%)	207 (32%)	242 (38%)	645 (100%)	
			% of Total	24%	25%	30%	79%	
	Total		% within Nationality	262 (32%)	281 (35%)	271 (33%)	814 (100%)	
			% of Total	32%	35%	33%	100%	
**Winter**	**Nationality**	**Non-Saudi**	% within Nationality	51 (37%)	50 (36%)	38 (27%)	139 (100%)	0.01
			% of Total	7.00%	7%	5%	19%	
		**Saudi**	% within Nationality	150 (25%)	174 (30%)	265 (45%)	589 (100%)	
			% of Total	21%	24%	36%	81%	
	Total		% within Nationality	201 (28%)	224 (31%)	303 (42%)	728 (100%)	
			% of Total	28%	31%	42%	100%	

**Table 7:** Linear regression of vitamin D stratified by age and nationality.

Coefficients ^a^	Model		Unstandardized Coefficients		Standardized Coefficients			95.0% Confidence Interval for B	
Seasons			B	Std. Error	Beta	T	Sig.	Lower Bound	Upper Bound
**Spring**	1	(Constant)	39	8.204		4.812	<0.001	22.169	56.788
		Age	−0.603	0.529	−0.242	−1.139	0.271	−1.72	0.514
		Nationality	−10.09	5.288	−0.405	−1.908	0.073	−21.247	1.067
**Summer**	1	(Constant)	36	2.517		14.221	<0.001	30.838	40.759
		Age	−0.43	0.154	−0.188	−2.796	<0.001	−0.733	−0.127
		Nationality	−1.316	2.001	−0.044	−0.658	0.511	−5.26	2.627
**Autumn**	1	(Constant)	33	1.387		23.968	<0.001	30.52	35.965
		Age	−0.417	0.085	−0.185	−4.902	<0.001	−0.584	−0.25
		Nationality	0.613	1.152	0.02	0.532	0.595	−1.648	2.875
**Winter**	1	(Constant)	37	1.574		23.747	<0.001	34.278	40.457
		Age	−0.729	0.091	−0.3	−7.988	<0.001	−0.908	−0.55
		Nationality	0.265	1.208	0.008	0.22	0.826	−2.106	2.636

^a^ Dependent variable: total 25(OH) vitamin D. Being of Saudi nationality negatively, though not significantly, affected the circulating levels of 25(OH) vitamin D in all seasons.

**Table 8:** Regression analysis of the 25(OH) vitamin D level stratified by gender.

Variables in the Equation									95% C.I. for EXP (B)	
	Season of Sample Collection		B	S.E.	Wald	df	Sig.	Exp (B)	Lower	Upper
**Autumn**	Step 1 ^a^	Gender	0.513	0.17	9.082	1	0.003	1.670	1.196	2.332
		Constant	−0.876	0.088	98.582	1	<0.001	0.416		
**Spring**	Step 1 ^a^	Gender	0	1.291	0	1	1.000	1.000	0.08	12.557
		Constant	−1.099	0.577	3.621	1	0.057	0.333		
**Summer**	Step 1 ^a^	Gender	0.086	0.296	0.084	1	0.772	1.090	0.61	1.947
		Constant	−0.528	0.164	10.33	1	0.001	0.590		
**Winter**	Step 1 ^a^	Gender	0.686	0.185	13.763	1	<0.001	1.986	1.382	2.853
		Constant	−1.145	0.099	133.213	1	<0.001	0.318		

^a^ Variable(s) entered on step 1: gender.

**Table 9:** Seasonal comparisons of the mean serum concentrations of 25(OH)D between age groups.

		Means of 25(OH)D Concentrations (ng/mL)			
Age Categories	N (Frequency)	Autumn	Winter	Spring	Summer
**0–1**	5	61	71	0	64
**2–5**	117	39	43	66	36
**6–11**	247	27	28	22	29
**12–18**	434	24	24	20	26
**19–21**	987	26	24	29	28
Total	1790	27	25	25	29
***p*-Value**		*p* < 0.001	*p* < 0.001	*p* < 0.001	0.001

## Data Availability

The data are available upon request due to restrictions on privacy or ethical issues. The data presented in this study are available upon request from the corresponding author. The data are not publicly available due to confidentiality and ethical issues.
